# Study of the Thermal Properties and the Fire Performance of Flame Retardant-Organic PCM in Bulk Form

**DOI:** 10.3390/ma11010117

**Published:** 2018-01-12

**Authors:** Anabel Palacios, Alvaro De Gracia, Laia Haurie, Luisa F. Cabeza, A. Inés Fernández, Camila Barreneche

**Affiliations:** 1 Department of Materials Science & Physical Chemistry, Universitat de Barcelona, Martí i Franqués 1, 08028 Barcelona, Spain; apalacios@ub.edu (A.P.); ana_inesfernandez@ub.edu (A.I.F.); 2 Departament d’Enginyeria Mecanica, Universitat Rovira i Virgili, Av. Paisos Catalans 26, 43007 Tarragona, Spain; alvaro.degracia@urv.cat; 3 Departament de Tecnologia de l’Arquitectura, Universitat Politècnica de Catalunya, Av. Dr. Marañon 44-50, 08028 Barcelona, Spain; laia.haurie@upc.edu; 4 GREA Innovació Concurrent, Universitat de Lleida, Edifici CREA, Pere de Cabrera s/n, 25001 Lleida, Spain; lcabeza@diei.udl.cat

**Keywords:** phase change materials (PCMs), thermal energy storage (TES), flame retardants, dripping test, differential scanning calorimetry (DSC)

## Abstract

The implementation of organic phase change materials (PCMs) in several applications such as heating and cooling or building comfort is an important target in thermal energy storage (TES). However, one of the major drawbacks of organic PCMs implementation is flammability. The addition of flame retardants to PCMs or shape-stabilized PCMs is one of the approaches to address this problem and improve their final deployment in the building material sector. In this study, the most common organic PCM, Paraffin RT-21, and fatty acids mixtures of capric acid (CA), myristic acid (MA), and palmitic acid (PA) in bulk, were tested to improve their fire reaction. Several flame retardants, such as ammonium phosphate, melamine phosphate, hydromagnesite, magnesium hydroxide, and aluminum hydroxide, were tested. The properties of the improved PCM with flame retardants were characterized by thermogravimetric analyses (TGA), the dripping test, and differential scanning calorimetry (DSC). The results for the dripping test show that fire retardancy was considerably enhanced by the addition of hydromagnesite (50 wt %) and magnesium hydroxide (50 wt %) in fatty acids mixtures. This will help the final implementation of these enhanced PCMs in building sector. The influence of the addition of flame retardants on the melting enthalpy and temperatures of PCMs has been evaluated.

## 1. Introduction

In recent years, governments have started to be more aware of the urgent need to make better use of the world’s energy resources. Total energy consumption in buildings (around 34% [1]) has become a focus of studies and publications, with the aim of improving energy efficiency and reducing greenhouse gas emissions. Other than ameliorate the situation by reducing energy consumption and improving the efficiency of appliances, etc., the building envelopes must be more energy efficient as well, and thermal energy storage (TES) is one promising way to improve energy efficiency. Phase-change materials (PCMs) incorporated in the building walls can smooth temperature fluctuations and reduce electrical consumption by restricting the necessity to use electricity for heating/cooling during peak load periods [2,3].

Phase change materials (PCMs) have received much attention within the scientific community due to the fact that they present high-energy storage density when used in the required range of working temperatures. The implementation of organic PCMs in several applications such as heating and cooling or improvement of indoor building comfort is an important target in (TES) [4]. However, one of the major drawbacks of organic PCM implementation is flammability. The addition of flame retardants to PCMs or shape-stabilized PCMs is one of the approaches to address this problem and improve their final deployment as building materials.

The mechanism of action of PCMs is described as follows: when the temperature reaches the melting temperature they change their state from solid to liquid, being able to store large amounts of energy, and the same happens during the reverse process, but in this case the heat is released. As a result, PCMs in active systems provide high thermal energy storage capacity for the building component as a ventilated façade [5], or active slab, which might be used as storage in a building’s active system [5] or to improve thermal inertia as passive systems inside buildings act as thermal regulators [6,7]. 

**Figure 1:**
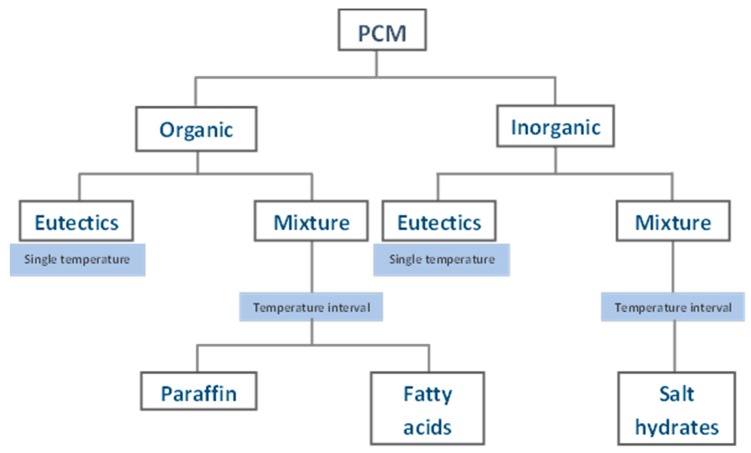
PCM classification adapted from Abhat [8] regarding the PCM type available within the temperature range of 20 to 30 °C.

There are several ways to classify PCMs, according to their latent heat of fusion and melting point, according to temperature gradients [4] and, most commonly, according to the method used to exchange heat and to change the state, which was used by Abhat [8] in 1983. The most used PCM types are the one shown in Figure 1 within the temperatures range of this study (between 20 and 30 °C). 

The mainly studied organic PCMs are paraffin and fatty acids. As regards inorganic PCMs, salt hydrates are the most used. Cabeza et al. reported a comparison of advantages and disadvantages of organic and inorganic PCMs [4]. Inorganic PCMs present several drawbacks that complicate their use [9]. Drawbacks are sub-cooling, delay of the solidification process as well as capacity of store heat, corrosion and segregation. On the other hand, in general terms, organic PCMs represent an adequate option to be used in TES, accomplishing requirements such as chemical and thermal stability, no corrosion and low or no sub-cooling, which is an important topic in order to endure many cycles [10]. However, those substances also have problems such as lower phase change enthalpy, high flammability, and lower thermal conductivity than salt hydrates [11], but this can be minimized with the use of additives. Overall, the main drawbacks of inorganic PCMs are difficult to solve with the addition of additives. However, the addition of flame retardants or conductive particles could be a successful strategy to overcome the main drawbacks of organic PCMs.

In order to solve one of the major problems of organic PCM implementation in the buildings sector—flammability—several studies have been published [12,13,14,15,16,17]. Cai et al. [12] investigated the effect of adding expanded graphite (EG) and ammonium polyphosphate (APP) to PCM composites based on paraffin with a high-density polyethylene (HDPE) matrix. The results of thermogravimetric analysis (TGA) and cone calorimetry showed that EG and APP contributed to improving thermal stability properties and enhanced self-extinguishing properties of PCM composites. Moreover, thermal stability and flammability properties of palmitic acid/silicon dioxide (SiO_2_) composites were studied by Fang et al. [13], where these composites were prepared by using sol-gel methods. Therefore, palmitic acid was used as PCM, silicon dioxide acted as the supporting material and melamine was incorporated as a flame retardant. The results reported that melamine and a multi-porous material (SiO_2_) improve fire performance. In addition, Sittisart and Farid [14] studied a form-stable PCM consisting of paraffin (RT-21), high-density polyethylene (HDPE), and flame retardants such as magnesium hydroxide, aluminum hydroxide, expanded graphite (EG), ammonium polyphosphate (APP), pentaerythritol (PER), and treated montmorillonite (MMT), using a vertical burning test. They concluded that the addition of flame retardants improves thermal stability, and the best improvement in fire performance is showed by the mixture of APP+PER+MMT and APP+EG.

The present study is focused on how to reduce flammability of bulk-paraffin and bulk-fatty acid mixtures in liquid state without them being stabilized inside a matrix or encapsulated. To achieve this objective, organic PCMs were mixed with several flame retardants that act through different mechanisms to improve the fire performance of PCMs. The study of the effect of flame retardants directly on the bulk-PCM opens a new approach for improving fire performance of non-encapsulated PCMs. This will help the final implementation of these enhanced PCMs in the building sector. The main scope of the present work firmly connects fire-resistant performance with thermophysical properties.

## 2. Materials and Methods

### 2.1. Phase Change Materials

Different types of organic PCMs were tested in this study, namely paraffin and fatty acids. 

Thermophysical properties of paraffin have been investigated by many researchers [18,19,20,21]. There are several commercial paraffins, which present different melting points and heat of fusion. Paraffin RT-21 meets desirable melting temperature (21 °C) in order to be implemented to improve thermal comfort in buildings. This temperature range is related to the building standards of major countries in the south of Europe where the comfortable indoor temperature of buildings are defined [22]. 

On the other hand, fatty acids show some differences compared with paraffin: narrow range of phase change, addition of antioxidants to reach chemical stability, high heat of fusion and less change in volume during phase change. Fatty acid melting points range from −7 to 71 °C depending on their chemical structure, while melting enthalpy varies from 45 to 210 kJ/kg [18]. It is difficult to find a fatty acid that works in the thermal range required for building applications. Therefore, fatty acid eutectic mixtures provide an option to achieve proper phase change temperatures at close to 21 °C.

Paraffin RT-21 commercialized by Rubitherm [23] and fatty acid eutectic mixtures are described in Table 1. The fatty acids used to prepare the eutectic mixtures were provided by Panreac (Barcelona, Spain) and their purities are 98% for capric acid and 98.5% for myristic acid and palmitic acid. The temperature ranges of these PCMs are adequate for the established comfort temperatures for building applications. Paraffin shows low thermal conductivity (around 0.2 W m^−1^ °C^−1^), thermal and chemical stability, low volume changes during solidification process, and there are several different paraffinic PCMs suitable to be used to enhance thermal comfort in buildings. They are relatively cheap and are produced on a large scale [18].

### 2.2. Flame Retardants

Flame-retardant systems are proposed to delay or to inhibit combustion and there exist a large amount of flame retardants [24,25]. Nevertheless, the correct selection of the proper fire retardant is a key issue and not all of them will act properly for each PCM and application. Moreover, certain conditions are required in order to maintain the PCM properties. For example, the flame retardant has to be well dispersed and distributed in the PCM volume, and thermophysical PCM properties must remain constant. 

Depending on their nature, flame retardants can act in the condensed or in the gas phase through a physical or chemical process [24,25]. Flame retardants can be classified according to their mechanism of action in three categories:

- Gas phase flame retardants: The flame retardant interferes with the free radical combustion reaction. Halogenated flame retardants are an example of this category.
- Endothermic flame retardants undergo an endothermic decomposition in the range of temperatures at which combustion takes place. This endothermic reaction helps to withdraw heat from the substrate. Furthermore, these compounds evolve non-flammable gases such as water or CO_2_ that have a dilution effect. The metal oxide formed during the decomposition of a metal hydroxide form an insulating protective coating on the condensed phase.
- Char-forming flame retardants: In this case, the flame retardant promotes the formation of a protective coating on the flammable material that hinders the heat and oxygen transfer. Polyphosphates and intumescent flame retardants (IFRs) are among this category. IFRs consist of a combination of a carbon source, an acid source and a foaming agent. Usually, ammonium polyphosphate, pentaerythritol and melamine are the main ingredients of an IFR.

In this study, five different flame retardants have been used: three endothermic flame retardants (commercial grades of alminum and magnesium hydroxide, and non-commercial synthetic hydromagnesite already used in previous studies [26]), as well as two commercial char-forming flame retardants (ammonium polyphosphate and an IFR). Halogenated flame retardants were deliberately not selected due to their toxicity and environmental impact [25,27,28]. Table 2 summarizes onset decomposition temperature and enthalpy of decomposition of flame retardants used in this study.

### 2.3. Formulations

A matrix of formulations was tested in a preliminary study (see Table 3) in order to find the best performing flame retardant-PCM system. Several percentages of each flame retardant were tested to find the suitable flame-retardant load. The flame-retardant content required in order to reach a satisfactory fire performance depending on the nature and mechanism of action of the flame retardant was chosen and studied. According to the formulations used in the literature and recommended by the suppliers of polyolefin, it was decided to use at least 40% in weight for metal hydroxides and 15% for the char-forming flame retardants [29,30].

Samples containing 40 wt %, 50 wt % and 60 wt % of hydromagnesite and magnesium hydroxide in the PCM were tested as well as samples with 15 wt %, 20 wt % and 25 wt % of ammonium phosphate (APP) and IFR systems (Table 3). In the case of the paraffin bulk-PCM, the amount of APP used will be up to 40 wt % in order to evaluate the performance of high loads of flame retardant. The final formulations selected and characterized, named optimum, were those that gave lowest flammability with the lowest amount of fire retardant addition. Note that the lower the fire-retardant percentage the lower the change of PCM thermophysical properties, following the mixtures law [31].

## 3. Experimental Methods

### 3.1. Thermal Stability

Thermogravimetrical analysis (TGA) technique was used to characterize the thermal stability of the samples under study. Analyses were performed on the pure PCMs between 50 °C and 500 °C under N_2_ atmosphere with a flow of 80 mL/min at a heating rate of 10 °C/min with a mass of approximately 30 mg in a TA Instruments SDT Q600 (TA Instruments, Barcelona, Spain). 

### 3.2. Pyrolysis Combustion Flow Calorimeter (PCFC)

A pyrolysis combustion flow calorimeter [32] from Fire Testing Technology standardized according to ASTM D7309 [33] was used to evaluate the flammability of the PCM. The equipment consists of a pyrolysis chamber, where small samples, around 5 mg, are heated under nitrogen atmosphere up to 750 °C at 1 °C/s. The evolved gases are then transported by an inert gas to the combustor that works at 900 °C in a flow of 20 cm^3^ of oxygen and 80 cm^3^ of nitrogen. The heat release rate is calculated from the oxygen consumed by the evolved gases during their combustion in the combustor. 

### 3.3. Dripping Test

Flammability behavior was characterized by dripping test. The device is described in the Spanish standard UNE 23727 [34]. In this work the test was adapted to measure the properties of bulk PCM formulations. The dripping test is used to evaluate the flame and its propagation in a fire scenario.

PCM samples were weighted and introduced into a ceramic crucible, which was placed on a metallic grid 3 cm below a heat source of 500 W. Under this test conditions the heat flux on the surface of the samples was 3 W/cm^2^. The radiator was taken away and put back after each ignition and extinction. Three samples of each formulation were tested and the parameters determined were the time to ignition, the number of ignitions and the average time of flame persistence during the first 5 min of combustion. Temperature profiles reached during the dripping test were registered by a thermocouple.

Time to ignition (*t_i_*) is defined as the time at which the first flame appears on the surface of the sample. The average combustion time (*t_c_*) is related to the length of the combustion and, therefore, to the ability of the material to self-extinguish the flame once the heating source is removed. Finally, the number of ignitions is another parameter related to the fire reaction of the material. A combination of short combustion times together with numerous ignitions is a sign of a flammable material that is able to extinguish the flame if the heating source is removed. 

### 3.4. DSC

Samples (PCMs with fire retardant) were evaluated by Differential Scanning Calorimetry (DSC), which is a well-known technique to analyze the latent heat of PCM. Therefore, DSC is able to determine the enthalpy of fusion and solidification, as well as the phase change temperature of the analyzed material. Furthermore, the analysis was performed between *T_m_* ± 10 °C (being *T_m_* the PCM melting temperature) and 0.5 °C/min heating rate [35,36]. The amount of sample used was around 15 mg and the sample was placed into 40 mg aluminum crucibles, and the equipment used was a DSC 822e device supplied by Mettler Toledo (Barcelona, Spain). The experiment was tested under 50 mL min^−1^ N_2_ flow. In addition, the equipment precision is ±0.3 °C for temperature and ±3 kJ kg^−1^ for enthalpy results.

## 4. Results and Discussion

### 4.1. Thermal Stability 

**Figure 2:**
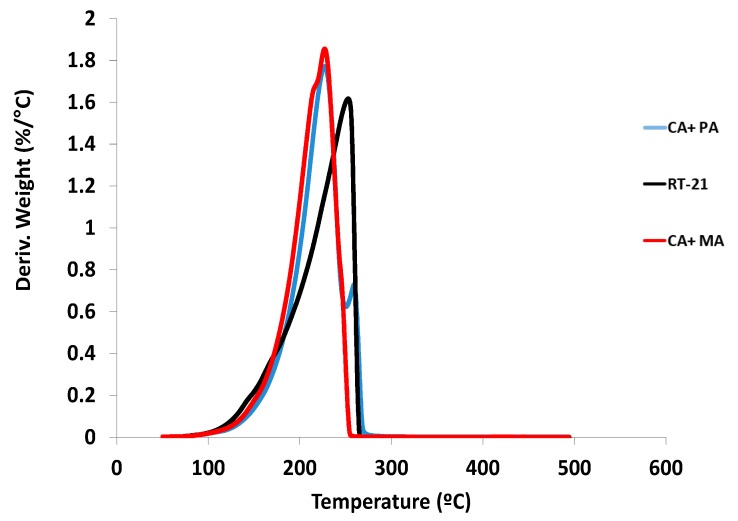
Derivative thermogravimetric curves of the PCM.

TGA results describe the thermal decomposition of the PCMs. As can be observed in Figure 2, paraffin decomposes in one step between 190 °C and 250 °C. In the mixture of capric and myristic acids, a shoulder can be distinguished before the main peak and the derivate of the TGA curve of the eutectic mixture of capric and palmitic acids exhibits two peaks. This behaviour can be attributed to the different decomposition temperature of the fatty acids in accordance with the chain length. 

### 4.2. PCFC

**Figure 3:**
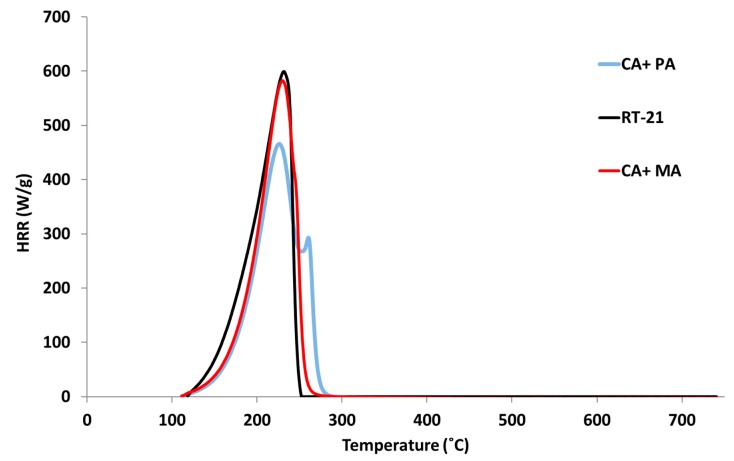
Heat released rate curves vs. temperature for organic PCM under study.

The curves of heat release rate (HRR) vs. temperature are shown in Figure 3. As can be observed, decomposition of the three PCM starts at around 100 °C. The peak of heat release rate (PHRR) corresponds to the maximum value of the HRR and the temperature of PHRR (T_PHRR_) is the temperature at which the PHRR takes place. High PHRR at low temperatures is usually related to a higher contribution in case of fire. In this case, all the samples have a similar T_PHRR_: 231 °C for the paraffin, 230 °C for the eutectic mixture of caprilic and palmitic fatty acids, and 226 °C for the eutectic mixture of caprilic and myristic acids. Regarding the PHRR, the paraffin PCM shows one unique peak of approx. 600 W/g. The CA + MA PCM shows one peak of 583 W/g and a shoulder can be identified around 245 °C. The PHRR of the CA + PA mixture is significantly lower, at 466 W/g, and a smaller second peak (292 W/g) takes place at 258 °C. 

### 4.3. Dripping Test

As mentioned before, the preliminary study allowed us to select some of the percentages of flame retardant based on fire performance. Taking into account the formulations proposed in Table 3, the formulations with the lowest amount of each fire retardant that behaves with the best flammability performance are highlighted in Table 4. 

Moreover, Table 4 provides the most important parameters in the dripping test: time to ignition (*t_i_*), the number of ignitions, and the average time of combustion (*t_C_*). 

An increase in the time to ignition is desirable, but it is not always obtained with the addition of flame retardants. This is due to the fact that many flame retardants act mainly on diminishing the heat release rate [37], which is believed to have more impact in case of a fire than ignitability [38]. 

The results for bulk paraffin PCMs demonstrate that the addition of 50% of endothermic flame retardants do not cause a significant improvement of the fire performance. The addition of char-forming flame retardants, APP and IFR, gives rise to a slight improvement. Paraffin PCM containing 20% of IFR increases the time to ignition, and two ignitions take place, which means that after the first ignition the sample self-extinguished the flame and it is necessary to approach the heating source again to produce the second ignition. This behaviour slightly reduces the average combustion time. The maximum percentage initially established for APP (range between 15 wt % and 25 wt %) was not enough to achieve fire inhibition in paraffin. For that reason, this percentage was increased up to 40 wt % in order to evaluate if higher loads could give rise to a better fire performance of paraffin. In the case of paraffin with 40 wt % of APP, ignitability is not improved, as can be noticed by the reduction in the time to ignition. However, the combination of PCMs and 40 wt % APP shows a clear ability to extinguish the flame, which is supported by the reduction in the average combustion time as well as the number of ignitions.

The self-extinguishing ability is much clear in some of the fatty acid flame retarded systems. Especially, magnesium hydroxide and hydromagnesite are remarkably good for reducing the combustion time and therefore also increase the number of ignitions. These two endothermic flame retardants exhibit their thermal decomposition at higher temperatures than aluminum hydroxide, which does not produce a remarkable effect on the fire performance. Probably in the dripping test the release of flammable gases is produced in a broad range of temperatures and therefore, flame retardants that act at higher temperatures or in a wider range of temperatures are favored. The char-forming flame retardants in the loads used (20% in weight) do not lead to a successful formation of protective char and, therefore, the fire performance of the PCMs is not improved.

In summary, the best performing formulation for each PCM-flame-retardant combination based on dripping test results that are highlighted in grey in Table 4 are: the addition of 50 wt % of hydromagnesite and magnesium hydroxide for fatty acid mixtures, and 40 wt % of APP slightly contributing to improve the fire reaction of paraffin. Aluminum hydroxide does not perform properly with the selected PCM.

**Figure 4:**
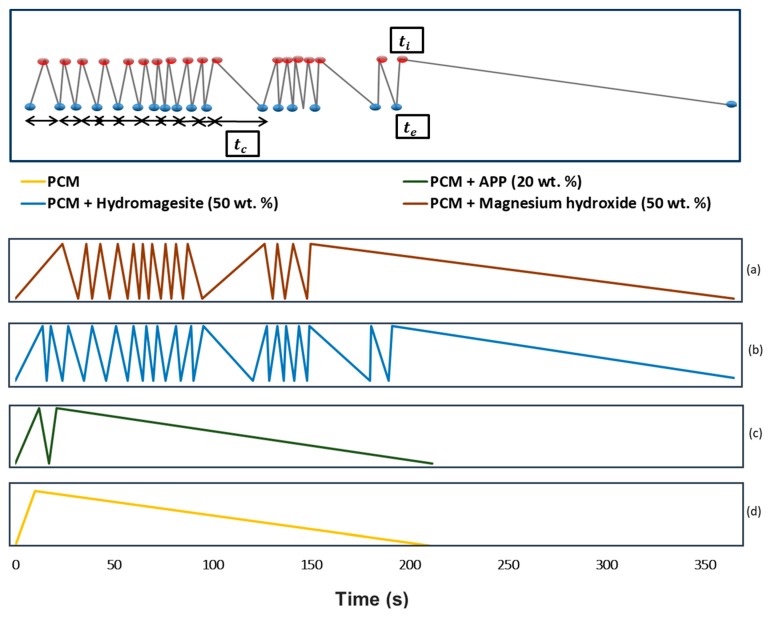
Legend of the parameters to comprehend the graph (above) and ignition-extinction periods for CA + MA mixture formulation with flame retardants (below). (**a**) Fire performance of capric and myristic eutectic with 50 wt % magnesium hydroxide; (**b**) Fire performance of capric and myristic eutectic with 50 wt % hydromagnesite; (**c**) Fire performance of capric and myristic eutectic with 20 wt % APP; (**d**) Fire performance of capric and myristic eutectic mixture. *t_i_*: ignition time; *t_e_*: extinction time; *t_c_*: combustion time.

Figure 4 illustrates ignition/extinction periods obtained for the dripping test for capric acid and myristic acid eutectic mixture, for example. The peaks and valleys show the time when the PCM ignites (*t_i_*) (up) and extinguishes (*t_e_*) (down). Furthermore, the period of time between each ignition and extinction is the combustion time (*t_c_*). Figure 4d shows the case of the plain PCMs. It can be seen that once ignited, the PCM keeps on burning until the fuel is exhausted. The addition of APP as flame retardant does not introduce significant changes in the fire performance (Figure 4c), from which we can conclude that this flame retardant is not acting on the load and under the conditions applied. The elevated number of peaks in Figure 4a,b indicates that a high number of ignitions and extinctions occur during the test. This fact is related to the ability to extinguish the flame of the PCM formulations with magnesium hydroxide and hydromagnesite. In the case of magnesium hydroxide, a delay on the time to ignition is also observed.

### 4.4. Thermal Characterization

Thermophysical property results obtained by DSC are listed in Table 5. As expected, DSC results show how the PCM thermophysical properties are affected when fire retardants are added to the formulation.

Paraffin melting temperature and enthalpy were not affected by the addition of 40 wt % APP. 

On the other hand, fatty acid mixtures showed a clear reduction of the melting enthalpy, by a factor of 3 when 50 wt % flame retardant is added, while the melting temperature remained almost equal to that of the single PCMs.

### 4.5. Further Work

In this paper, the thermal enthalpy of the different PCM systems has been determined. In order to obtain more information about the thermal performance of each PCM formulation, it would be interesting to evaluate their thermal inertia. In this case, the experiment developed must allow performing temperature/time curves of bulk PCMs with a control of the heat flow and measurements of the peak temperature under temperature oscillation. This kind of equipment is not commonly found [39] and therefore the authors have not been able to carry out these tests. 

An alternative to direct measurement of thermal inertia is to determine an effective thermal inertia. A measurement procedure is described in the standard ASTM E 1321-Standard Test Method for determining material ignition and flame spread properties [40]. The dripping test used in this paper is not suitable for this kind of experiment, but some other small-scale radiant exposure tests that allow controlling and modifying the heat flux could be used to obtain effective thermal properties [41]. 

## 5. Conclusions

The effect of different types of flame retardants such as APP, MPP, hydromagnesite, magnesium hydroxide and aluminum hydroxide on PCMs was investigated. Fire performance of paraffin was not significantly improved by any of the flame retardants used. Nevertheless, 40 wt % APP slightly contributed to enhance the self-extinguishing ability of paraffin PCMs.

On the other hand, the considered fatty acid mixtures showed a remarkable improvement in the fire reaction when loadings of 50 wt % of hydromagnesite or magnesium hydroxide were added as flame retardants. Nevertheless, aluminum hydroxide, also an endothermic flame retardant, did not show any positive effect on the fatty acids. This fact could be explained due to the lower decomposition temperature of aluminum hydroxide compared with the other two endothermic flame retardants. The char-forming flame retardants did not improve the fire performance of the fatty acids.

Deeply analyzing the fire performance and thermal stability of organic PCM-flame-retardant mixture in bulk, it can be concluded that for fatty acids the enthalpy of fusion decreased following the mixtures law by the addition of flame retardants, but for paraffin melting enthalpy did not decrease. On the other hand, the phase change temperature remains equal.

In summary, magnesium hydroxide and hydromagnesite strongly affect the melting enthalpy and tend to decrease the melting temperature, while APP has a much lower influence on melting enthalpy and temperature when added to the bulk PCMs. The addition of flame retardants will decrease the flammability of bulk-organic PCMs, but more research is needed to perform a fire-retardant screening in order identify new fire retardants that would work with less quantity to keep the thermophysical properties of PCMs as high as possible to ensure their applicability.

## Figures and Tables

**Table 1:** Properties of PCM used on this study (from literature).

Compound	Melting Temperature (°C)	Heat of Fusion (kJ/kg)	Thermal Conductivity (W/m·°C)
Paraffin RT-21	21 [23]	100 [23]	0.2 [23]
73.5% Capric acid + 26.5% Myristic acid	24.1 [4]	152 [4]	n.a
75.2% Capric acid + 24.8% Palmitic acid	22.1 [4]	153 [4]	n.a

**Table 2:** Properties of tested flame retardant [24,29].

Compound	Method	Onset Decomposition Temperature (°C)	Enthalpy of Decomposition (kJ/kg)
Aluminum hydroxide	Endothermic decomposition	180	1300
Magnesium hydroxide	Endothermic decomposition	340	1450
Hydromagnesite	Endothermic decomposition	200	800
APP	Char forming	190	-
IFR	Char forming	190	-

**Table 3:** Flame retardant formulations.

Formulations
PCM + (15%-20%-25%-40%) APP
PCM + (40%-50%-60%) Hydromagnesite
PCM + (40%-50%-60%) Magnesium hydroxide
PCM + (40%-50%-60%) Aluminum hydroxide
PCM + (15%-20%-25%) IFR

**Table 4:** Dripping test results for PCM.

Flame Retardant (wt %)	Ignition Time (s)	N° of Ignitions	Average Combustion Time (s)
**Paraffin RT-21**	26	1	300
60% Paraffin + 40% APP	20	3	82
50% Paraffin + 50% HM	26	1	293
60% Paraffin + 20% IFR	50	2	239
50% Paraffin + 50% Al(OH)_3_	27	1	288
50% Paraffin + 50% Mg(OH)_2_	26	1	286
**CA + MA**	19	1	200
80% (CA + MA) + 20% APP	12	2	109
50% (CA + MA) + 50% HM	14	15	8
80% (CA + MA) + 20% IFR	21	2	102
50% (CA + MA) + 50% Al(OH)_3_	19	2	117
50% (CA + MA) + 50% Mg(OH)_2_	24	17	4
**CA + PA**	12	1	224
80% (CA + PA) + 20% APP	10	2	104
50% (CA + PA) + 50% HM	9	17	7
80% (CA + PA) + 20% IFR	16	3	106
50% (CA + PA) + 50% Al(OH)_3_	14	1	322
50% (CA + PA) + 50% Mg(OH)_2_	32	26	4

**Table 5:** DSC of PCM-fire retardant optimum combination results.

	Compositions	Melting Enthalpy (kJ/kg)	Peak Temperature (°C)
**PCMs**	Paraffin RT-21	118 ± 3	22.3 ± 0.2
	73.5% Capric acid + 26.5% Myristic acid	143 ± 3	24.1 ± 0.2
	75.2% Capric acid + 24.8% Palmitic acid	141 ± 3	23.3 ± 0.2
**PCM + Flame Retardant**	60% Paraffin RT-21 + 40% APP	111 ± 4	22.6 ± 0.2
	50% CA + MA + 50% Hydromagnesite	53 ± 3	22.0 ± 0.2
	50% CA + MA + 50% Magnesium hydroxide	55 ± 3	24.4 ± 0.2
	50% CA+PA + 50% Hydromagnesite	56 ± 1	19.0 ± 0.6
	50% CA+PA + 50% Magnesium hydroxide	55 ± 2	23.0 ± 0.2
